# A protecting group strategy to access stable lacunary polyoxomolybdates for introducing multinuclear metal clusters†

**DOI:** 10.1039/d0sc06133f

**Published:** 2021-01-13

**Authors:** Chifeng Li, Atsuhiro Jimbo, Kazuya Yamaguchi, Kosuke Suzuki

**Affiliations:** Department of Applied Chemistry, School of Engineering, The University of Tokyo 7-3-1 Hongo, Bunkyo-ku Tokyo 113-8656 Japan ksuzuki@appchem.t.u-tokyo.ac.jp kyama@appchem.t.u-tokyo.ac.jp; Precursory Research for Embryonic Science and Technology (PRESTO), Japan Science and Technology Agency (JST) 4-1-8 Honcho, Kawaguchi Saitama 332-0012 Japan

## Abstract

Although metal-containing polyoxomolybdates (molybdenum oxide clusters) exhibit outstanding catalytic properties, their precise synthetic method has not yet been developed. This is mainly because the very low stability of the multivacant lacunary polyoxomolybdates limited their use as synthetic precursors. Here, we present a “protecting group strategy” in polyoxometalate synthesis and successfully develop an efficient method for synthesising multinuclear metal-containing polyoxomolybdates using pyridine as a protecting group for unstable trivacant lacunary Keggin-type polyoxomolybdate [PMo_9_O_34_]^9−^. Specifically, tetranuclear cubane- and planar-type manganese clusters were selectively synthesised in the polyoxomolybdates using the present method. The importance of this work is that, in addition to being the first practical way of utilizing multivacant lacunary polyoxomolybdates as precursors, this new “protecting group strategy” will make it possible to produce polyoxometalates with unexplored structures and properties.

## Abstract 2

A “protecting group strategy” for unstable lacunary polyoxomolybdates enabled successful synthesis of two types of tetranuclear manganese clusters.

## Graphical abstract

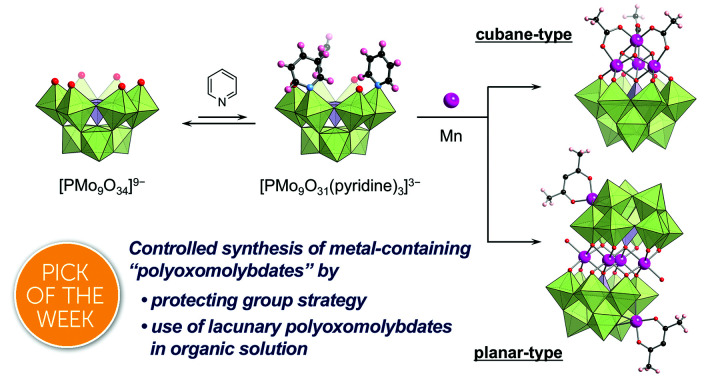

## Introduction

Polyoxometalates (POMs) are attractive nanosized anionic metal oxide clusters that exhibit large structural diversity and have found applications in various fields including catalysis, analytical chemistry, medicine and optical materials.^1,2^ Their properties, such as redox potentials and acidities, can be finely controlled by selecting their structures, constituent elements and oxidation states. In addition, the introduction of transition metals into POMs allows control of these properties and/or achieve synergetic properties that cannot be realised by POM skeletons alone. Among POMs, “polyoxomolybdates” (molybdenum oxide clusters) showed outstanding catalytic and electrochemical properties.^3^ Typically, the catalytic activities of polyoxomolybdates in oxidation reactions are superior to those of tungsten-based analogues. For example, vanadium-containing polyoxomolybdates exhibited higher catalytic activity for oxidative transformations of various organic compounds than their corresponding polyoxotungstates.^4^ The partial doping of Mo atoms into Weakley-type cobalt-containing polyoxotungstates can greatly improve their catalytic properties in water oxidation reaction.^5^

**Fig. 1:**
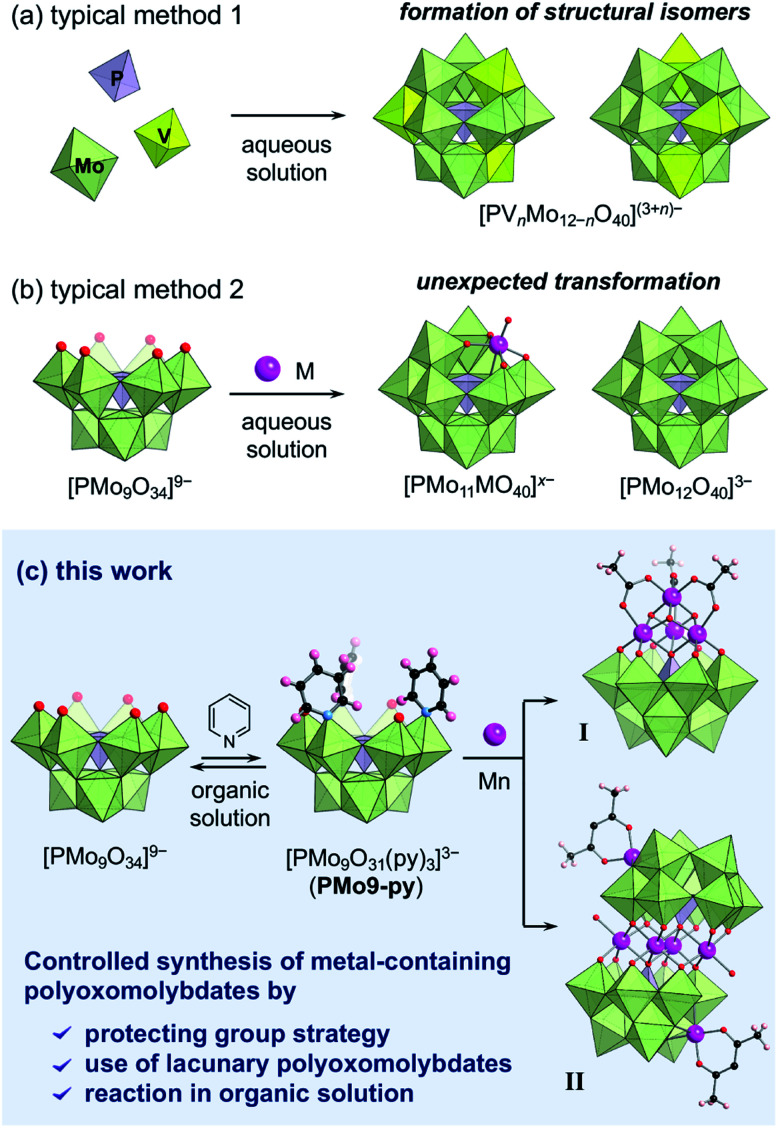
Schematic diagram of the synthetic method of metal-containing polyoxomolybdates: (a) conventional one-step synthesis of phosphovanadomolybdates; (b) reaction of trivacant lacunary polyoxomolybdates and metal cations in aqueous solution. (c) This work: protecting group strategy for the selective synthesis of metal-containing polyoxomolybdates using a pyridine-protected trivacant lacunary polyoxomolybdate in organic solution.

The controlled synthesis of multinuclear transition metal-containing “polyoxotungstates” has been achieved using multivacant lacunary “polyoxotungstates” as inorganic ligands (or templates).^6^ For example, our group has developed a powerful method for the use of metastable multivacant lacunary polyoxotungstates in organic solutions. By introducing metal atoms into lacunary POMs in a controlled manner, we synthesised various metal-containing polyoxotungstates with unique catalytic, photocatalytic and magnetic properties.^7^ While considerable efforts have been made in POM synthesis, the precise synthesis of multinuclear transition metal-containing “polyoxomolybdates” has not yet been achieved. Typically, metal-containing polyoxomolybdates have been synthesised by the one-step reaction of Mo atoms, heteroatoms (*e.g.* P, Si, Ge) and target metals (*e.g.* V) in aqueous solution (Fig. 1a).^8^ However, using this method, several structural isomers were formed, and it is difficult to control the position and number of introduced metals in polyoxomolybdates. Because lacunary polyoxomolybdates are extremely unstable, the reaction of multivacant lacunary polyoxomolybdates in aqueous solution typically results in an unexpected transformation into monovacant structures or fully occupied Keggin and Wells–Dawson type structures (Fig. 1b).^2*e*,6*b*,9^ Therefore, the development of synthetic method for the controlled introduction of transition metals into lacunary polyoxomolybdates is undoubtedly both challenging and highly demanding.

Recently, we determined that the metastable Keggin-type trivacant lacunary phosphomolybdate can be considerably stabilised by simply coordinating pyridine molecules at the vacant sites, and it can be isolated as [PMo_9_O_31_(py)_3_]^3−^ (py = pyridyl, **PMo9-py**).^10^ The pyridyl groups can protect the highly reactive vacant sites and suppress the unexpected structure transformation and dimerisation of lacunary species. Notably, the introduced pyridyl groups are moderately labile; therefore, **PMo9-py** can be used as a precursor for the synthesis of POM–organic hybrid architectures. Based on these results, here we envisioned that **PMo9-py** is a promising precursor for the controlled synthesis of metal-containing polyoxomolybdates.

In this study, we present a “protecting group strategy” in POM synthesis and establish methods for the synthesis of multinuclear metal clusters using the pyridine-protected A-α-Keggin-type trivacant lacunary polyoxomolybdate **PMo9-py** as a precursor. By reacting **PMo9-py** with manganese species in organic solution, two types of multinuclear manganese clusters [*i.e.* tetranuclear cubane- (**I**) and a planar-type (**II**) manganese clusters] were selectively synthesised (Fig. 1c). This method fully utilised **PMo9-py** as a well-defined precursor and performed the synthesis in organic solution, which avoided the complex equilibria in aqueous media. Pyridine molecules acted as protecting groups of unstable lacunary polyoxomolybdates for the selective introduction of multinuclear metal clusters. To our knowledge, this is the first report of a successful synthesis of multinuclear transition metal clusters using multivacant lacunary polyoxomolybdates.

## Results and discussion

### Synthesis of a tetranuclear cubane-type manganese cluster

**Fig. 2:**
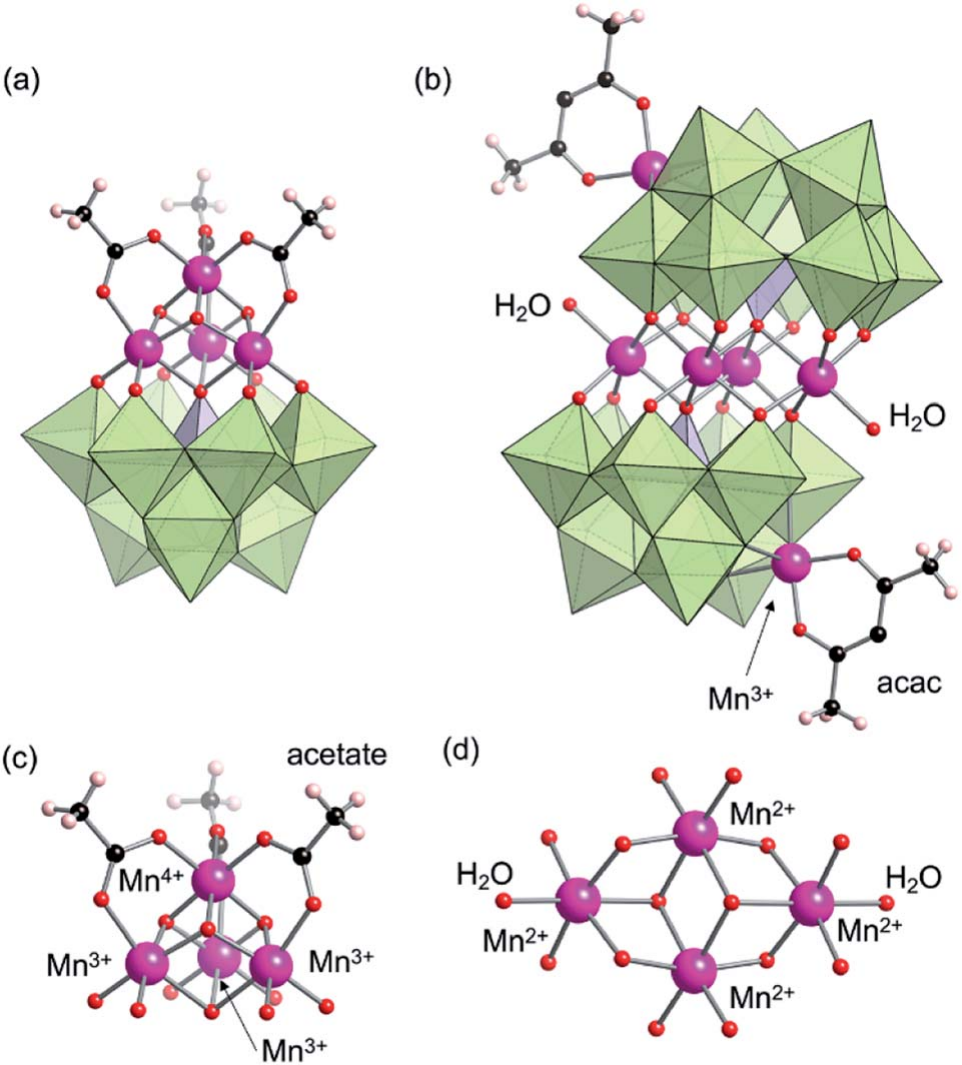
Crystal structures of the anion parts of (a) **I** and (b) **II**. The bottom figures are the selected views of manganese oxide clusters of (c) **I** and (d) **II**. Magenta, black, pink and red spheres represent Mn, C, H and O atoms, respectively; light green and light purple polyhedra represent {MoO_6_} and {PO_4_}, respectively.

First, we investigated the reaction of trivacant lacunary polyoxomolybdate TPP_3_H_6_[PMo_9_O_34_] (TPP = tetraphenylphosphonium)^10^ and Mn(OAc)_3_ (OAc = acetate) in organic solution such as acetonitrile and acetone. However, owing to its low stability even in organic solution, cold-spray ionisation (CSI)-mass spectrometry revealed that the structure transformation of [PMo_9_O_34_]^9−^ promptly proceeded to form a complex mixture including a fully occupied Keggin-type structure [PMo_12_O_40_]^3−^, a mononuclear manganese-containing structure [PMo_11_MnO_39_]^4−^, and other unidentified species (Fig S1a†). In contrast, when the pyridine-coordinating trivacant lacunary Keggin-type polyoxomolybdate [PMo_9_O_31_(py)_3_]^3−^ (**PMo9-py**) and Mn(OAc)_3_ were reacted in a mixture of dichloromethane and acetonitrile (1/1, v/v) at 0 °C for 2 h, the CSI-mass spectrum showed a set of signals centred at *m*/*z* = 2902.9 that were assignable to [TPP_3_H_1_PMo_9_O_37_Mn_4_(CH_3_COO)_3_]^−^ (theoretical *m*/*z*: 2902.7, Fig. S1b†), which supported the formation of the cubane-type structure **I**. Additionally, brown crystals of **I** suitable for crystallographic analysis were obtained from the solution (see ESI† for details). The X-ray crystallographic analysis revealed that the anion structure of **I** was a monomeric structure that consisted of four Mn atoms, where three Mn atoms occupied the vacant sites of a [B-α-PMo_9_O_34_]^9−^ unit, and the other Mn atom was stranded on the top of three Mn atoms (Fig. 2a, S2 and Tables S1–S3†). These Mn atoms were bridged by three acetate ligands, which formed a {Mn_4_O_3_(OAc)_3_} cubane-like core.^11^ These results showed that pyridyl ligands successfully acted as protecting groups for the unstable lacunary polyoxomolybdate and they prevented the structure transformation of lacunary polyoxomolybdate frameworks. Although cubane-type structures in Dawson- and Keggin-type polyoxotungstates as well as polyoxovanadates have been reported,^12^ this is the first report of cubane-type structure in polyoxomolybdates.

**Fig. 3:**
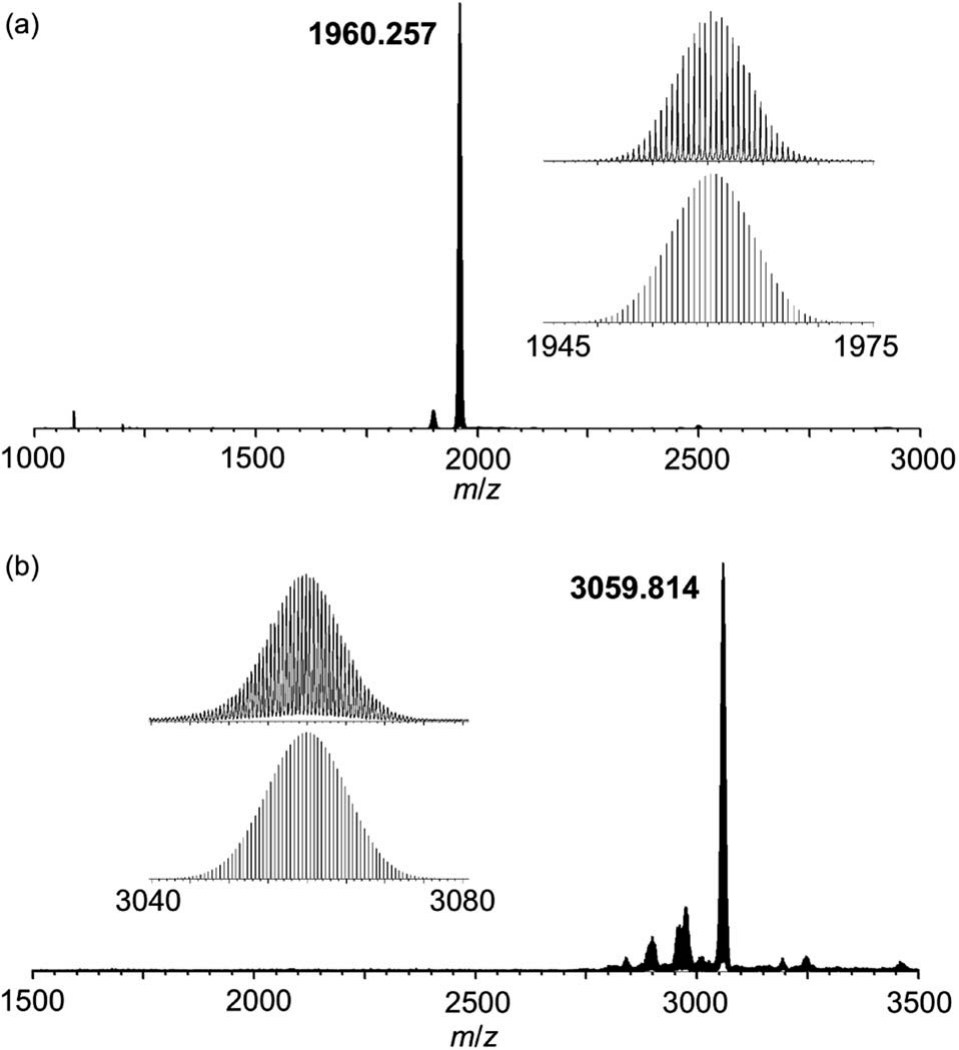
Positive-ion ESI-mass spectra of (a) **I** and (b) **II** in acetonitrile. Insets: (a) spectrum in the range of *m*/*z* 1945–1975, and simulated patterns for [TPP_6_HPMo_9_O_37_Mn_4_(OAc)_3_]^2+^ (*m*/*z*: 1960.262); (b) spectrum in the range of *m*/*z* 3040–3080 and simulated patterns for [TPP_8_{Mn(acac)}_2_Mn_4_(PMo_9_O_34_)_2_]^2+^ (*m*/*z*: 3059.837).

The bond valence sum (BVS) values of the three Mn atoms on the vacant sites were in the range of 3.03–3.05, while that of the Mn atom on the top was 4.02, which showed the mixed-valence cubane-type structure {Mn^3+^_3_Mn^4+^O_3_(OAc)_3_} (Fig. 2c and Table S2†). The BVS values of Mo atoms (6.00–6.17) and P atom (4.95) revealed that their valences were +6 and +5, respectively. The Mn^4+^ atom was likely formed by aerobic oxidation during the reaction in air. The average distance between three Mn^3+^ atoms and Mn^4+^ atom at the top is 2.787 Å (Table S3†). Considering that the distances in previously reported cubane-type structures in polyoxotungstates were in the range of 2.801–2.837 Å, Mn^4+^ at the top of the core of **I** is slightly more closely positioned to the {PMo_9_} unit (Table S6†). The electrospray ionisation (ESI)-mass spectrum of **I** in acetonitrile showed signal sets centred at *m*/*z* = 1960.257, which were assignable to [TPP_6_HPMo_9_O_37_Mn_4_(OAc)_3_]^2+^ (theoretical *m*/*z*: 1960.262, Fig. 3a). The ESI-mass spectrum indicated that the anion structure of **I** was stable and remained in solution. By combining the X-ray crystallographic analysis, ESI mass spectrum, cyclic voltammogram, TG analysis (Fig. S4a†) and elemental analysis, the formula of **I** was revealed as TPP_3_H_2_[Mn^3+^_3_Mn^4+^O_3_(OAc)_3_(B-α-PMo_9_O_34_)]·(H_2_O)·(CH_3_CN).

### Synthesis of a tetranuclear planar-type manganese cluster

Besides the cubane-type structure, another important structure in multinuclear transition metal-containing POMs is the Weakley-type structure with planar-type tetranuclear cores.^1,2,13^ During the formation of the structure of **I**, bridging acetate ligands may have prevented the further dimerisation into a Weakley-type structure. Therefore, the type of ligands of manganese precursors may be essential in controlling the structures of products. As expected, we successfully synthesised a Weakley-type structure by reacting **PMo9-py** and Mn(acac)_2_ (acac = acetylacetonate) in 1,2-dichloroethane at 50 °C for 2 h (see ESI† for details). X-ray crystallographic analysis revealed that the anion structure of **II** consisted of six Mn atoms and two [B-α-PMo_9_O_34_]^9−^ units (Fig. 2b, S3, Tables S1, S4 and S5†). Four Mn atoms in a planar configuration formed a {Mn_4_O_14_(H_2_O)_2_} core and the core was sandwiched between two [B-α-PMo_9_O_34_]^9−^ units, which was identical to the Weakley-type structures.^14,15^ Two {Mn(acac)} units capped the edge of each [B-α-PMo_9_O_34_]^9−^ unit. The BVS of Mo atoms (5.96–6.08) and P atoms (4.70) indicated the respective valences of +6 and +5 (Table S4†). The BVS values of sandwiched tetranuclear Mn atoms were in the range of 2.04–2.09, which indicated that the valences of sandwiched tetranuclear Mn atoms were +2, which was similar to the case in other Mn-containing Weakley-type structures. The Mn atoms in two {Mn(acac)} units showed BVS values of 3.08 (Table S4†). These results indicated that manganese atoms in the capping {Mn(acac)} units were oxidised from Mn^2+^ to Mn^3+^ during the reaction, possibly by O_2_ in air. This conclusion is supported by the fact that when the reaction was performed under an Ar atmosphere, the crystals with a Weakley-type structure were not obtained. The bond length of Mn2–O7 was 2.239 Å, which was slightly longer than that of other similar structures (Fig. 2c and Table S5†). According to BVS calculations, the two terminal O atoms on the sandwiched Mn atoms were aqua (H_2_O) ligands, judging by the BVS value of 0.28 (Fig. 2d and Table S4†). Although few Weakley-type polyoxomolybdates have been reported, all of them were synthesised by hydrothermal reactions of simple inorganic compounds;^15*a*,*b*,*c*^ thus, the use of multivacant lacunary polyoxomolybdates as precursors is unprecedented.

The ESI-mass spectrum of **II** in acetonitrile showed signal sets centred at *m*/*z* = 3059.814, which were assignable to [TPP_8_{Mn(acac)}_2_Mn_4_(PMo_9_O_34_)_2_]^2+^ (theoretical *m*/*z*: 3059.837, Fig. 3b). Two aqua ligands on the edge of the sandwiched tetranuclear Mn core were dissociated from the POM framework under the ESI-mass measurement conditions. The ESI-mass spectrum revealed that the anion structure of **II** was stable and remained in solution. Therefore, we investigated the electrochemical properties of **II**. The cyclic voltammogram (0.5 mM **II**, 100 mM TBAClO_4_ in CH_3_CN, TBA = tetra-*n*-butylammonium, Fig. S5b†) showed three pairs of quasi-reversible peaks, with oxidation peaks observed at 0.25, 0.50 and 0.88 V *versus* Ag/Ag^+^, respectively. These redox pairs can be assigned to Mn^2+^/Mn^3+^, Mn^2+^/Mn^3+^, and Mn^3+^/Mn^4+^ ([M(acac)]^2+^), respectively. By combining the X-ray crystallographic analysis, ESI-mass spectrum, cyclic voltammogram, TG analysis (Fig. S4b†) and elemental analysis, it was revealed that the formula of **II** was TPP_5.5_H_0.5_[{Mn^3+^(acac)}_2_Mn^2+^_4_(H_2_O)_2_(B-α-PMo_9_O_34_)_2_]·(C_2_H_4_Cl_2_)·3(H_2_O).

## Conclusions

In conclusion, we developed a new method of synthesising POMs by employing a “protecting group strategy” for unstable lacunary POMs. Specifically, using a pyridine-protected multivacant lacunary Keggin-type polyoxomolybdate as a precursor in organic solution, we overcame the low stability of lacunary polyoxomolybdates, which enabled the successful synthesis of two types of tetranuclear Mn clusters, *i.e.* cubane- (**I**) and planar-type (**II**) structures. These two Mn clusters possess structures that are similar to the previously reported structures on polyoxotungstates, which exhibited high catalytic activity in water oxidation or single-molecule magnetic properties.^10,11^ The synthetic method developed in this report represents the first practical way of utilising multivacant lacunary polyoxomolybdates as precursors. We believe that this new synthesis method will take POM synthesis to the next stage to produce POMs with unexplored structures and properties.

## Conflicts of interest

There are no conflicts to declare.

## Supplementary Material

SC-012-D0SC06133F-s001

SC-012-D0SC06133F-s002
