# Supplementary material for: A protecting group strategy to access stable lacunary polyoxomolybdates for introducing multinuclear metal clusters
Source: Chem Sci. 2021 Jan 13;12(4):1240–4. doi: 10.1039/d0sc06133f (PMC8179105; doi:10.1039/d0sc06133f)
Supplement: SC-012-D0SC06133F-s001 [file SC-012-D0SC06133F-s001.pdf]

Electronic Supplementary Material (ESI) for Chemical Science.
This journal is © The Royal Society of Chemistry 2020

Electronic Supplementary Information (ESI) for

A protecting group strategy to access stable lacunary polyoxomolybdates for introducing multinuclear metal clusters

Chifeng Li, Atsuhiro Jimbo, Kazuya Yamaguchi* and Kosuke Suzuki*

Instruments: Electrospray ionization mass (ESI-mass) spectra were recorded on a Waters Xevo G2-XS QTof instrument. Cold-spray ionization mass (CSI-mass) spectra were recorded on JEOL JMS-T100CS. IR spectra were measured on Jasco FT/IR-4100 using KBr disks. Thermogravimetric and differential thermal analyses (TG-DTA) were performed on Rigaku Thermo plus TG 8120. Inductively coupled plasma atomic emission spectroscopy (ICP-AES) analyses were performed on Shimadzu ICPS-8100. Elemental analyses for C, H and N were performed on Elementar vario MICRO cube at the Elemental Analysis Centre of the School of Science of the University of Tokyo. Cyclic voltammetric measurements were carried out with a Solartron SI 1287 Electrochemical Interface. A standard three-electrode arrangement was employed with a BAS glassy carbon disk electrode as the working electrode, a platinum wire as the counter electrode, and a silver wire electrode as the pseudoreference electrode. The voltage scan rate was set at 100 mV s^{-1}, and $TBAClO_4$ was used as an electrolyte. The potentials were measured using $Ag/AgNO_3$ reference electrode (10 mM $AgNO_3$, 100 mM $TBAClO_4$ in acetonitrile, 0.55 V vs NHE).

Materials: Acetonitrile (Kanto Chemical), dichloromethane (Kanto), 1,2-dichloroethane (Kanto Chemical), diethyl ether (Kanto Chemical), pyridine (**py**, Kanto Chemical), tetraphenylphosphonium bromide (TPPBr, TCI), manganese (III) acetate dihydrate (Merck Schuchardt) and manganese (II) acetylacetonate (Aldrich) were used as received. **PMo9-py** was synthesized according to our previous report.[S7]

X-ray crystallography: Diffraction measurements were made on a Rigaku VariMax Saturn 724 diffractometer with graphite monochromated Mo Kα radiation (λ = 0.71069 Å, 50 kV, 24 mA) at 123 K. The data were collected using CrystalClear and processed using CrysAlis^{Pro}.[S1] Neutral scattering factors were obtained from the standard source. In the reduction of data, Lorentz and polarization corrections were made. The structural analyses were performed using WinGX.[S2] All structures were solved by SHELXS-2013/1 (direct methods) and refined by SHELXL-2018/3.[S3] P, Mo, Mn and oxygen atoms in the POM frameworks, organic ligands (OAc and acac), and TPP cations were refined anisotropically. Some highly disordered TPP cations and solvent molecules were omitted by using SQUEEZE program.[S4] CCDC-2041768 and 2041769, contain the supplementary crystallographic data for **I** and **II**, respectively. These data can be obtained free of charge from The Cambridge Crystallographic Data Centre via www.ccdc.cam.ac.uk/data_request/cif.

BVS calculations: BVS values were calculated by the expression for the variation of the length r_{ij} of a bond

between two atoms i and j in observed crystal with valence V_i:

$$V_i = \sum_j \exp\left(\frac{r'_0 - r_{ij}}{B}\right)$$

where B is constant equal to 0.37 Å, r'_0 is bond valence parameter for a given atom pair.[S5,S6]

Synthesis and characterization of $[Mn^{3+}_3Mn^{4+}O_3(OAc)_3(B\text{-}\alpha\text{-}PMo_9O_{34})]^{5-}$ (I): To a mixture of acetonitrile and dichloromethane (1/1, v/v, 4 mL), **PMo9-py** (100 mg, 35.5 μmol) and $Mn(OAc)_3{\cdot}2H_2O$ (38.1 mg, 142 μmol, 4 equivalents with respect to **PMo9-py**) were added, and the resulting solution was stirred for 2 h at 0°C in 1 atm of air, followed by filtration through a membrane filter. After the reaction, by addition of excess amount of diethyl ether to the reaction solution, brown powder of **I** was obtained (103 mg, 83% yield based on **PMo9-py**). When the reaction solution was kept still at room temperature with the vessel open, the brown crystals of **I** suitable for X-ray crystallographic analysis were obtained after 1 week. Elemental analysis, calcd. (%) for $TPP_3H_2[PMo_9O_{37}Mn_4(OAc)_3]{\cdot}(H_2O){\cdot}(CH_3CN)$: C, 32.43; H, 2.59; N, 0.47; P, 4.18; Mn, 7.42; Mo, 29.15. Found: C, 31.99; H, 2.73; N, 0.32; P, 4.26; Mn, 7.57; Mo, 29.67. Positive-ion MS (ESI, acetonitrile): *m/z* 1960.257 (calcd. 1960.262 for $[TPP_6HPMo_9O_{37}Mn_4(OAc)_3]^{2+}$). IR (KBr pellet, cm^{-1}): 3417, 3065, 1634, 1587, 1563, 1558, 1483, 1437, 1394, 1343, 1188, 1109, 1065, 997, 946, 840, 796, 754, 722, 689, 626, 528, 383.

Synthesis and characterization of $[\{Mn^{3+}(acac)\}_2Mn^{2+}_4(H_2O)_2(B\text{-}\alpha\text{-}PMo_9O_{34})_2]^{6-}$ (II): To 1,2-dichloroethane (100 mL), **PMo9-py** (500 mg, 189 μmol) and $Mn(acac)_2$ (143 mg, 567 mmol, 3 equivalents with respect to **PMo9-py**) were added, and the resulting solution was stirred for 2 h at 50°C in 1 atm of air. Then, the resulting solution was filtered through a membrane filter. After diethyl ether (5.0 mL) was added, the filtrate was kept at 25°C. The brown crystals of **II** suitable for X-ray crystallographic analysis were obtained after 1 day (104 mg, 21% yield based on **PMo9-py**). Elemental analysis, calcd. (%) for $TPP_{5.5}H_{0.5}[\{Mn^{3+}(acac)\}_2Mn^{2+}_4(H_2O)_2(B\text{-}\alpha\text{-}PMo_9O_{34})_2]{\cdot}(C_2H_4Cl_2){\cdot}3(H_2O)$: C, 31.67; H, 2.56; P, 4.25; Mn, 6.04; Mo, 31.63. Found: C, 31.99; H, 2.73; N, 0.32; P, 4.37; Mn, 5.92; Mo, 31.44. Positive-ion MS (ESI, acetonitrile): *m/z* 3059.814 (calcd. 3059.837 for $[TPP_8\{Mn(acac)\}_2Mn_4(PMo_9O_{34})_2]^{2+}$). IR (KBr pellet, cm^{-1}): 3418, 2925, 2854, 2359, 1636, 1586, 1563, 1522, 1484, 1437, 1344, 1314, 1286, 1189, 1166, 1109, 1029, 998, 930, 875, 798, 723, 690, 636, 614, 587, 528.

Table S1. Crystallographic data of **I** and **II**.

compound name	**I**	**II**
molecular formula	$C_{108}H_{98}Mn_4Mo_9N_3O_{43}P_5$	$C_{174}H_{174}Cl_{20}Mn_6Mo_{18}O_{74}P_8$
Fw (g mol^{-1})	3363.96	6462.44
crystal system	triclinic	triclinic
space group	*P*-1 (No. 2)	*P*-1 (No. 2)
a (Å)	16.1432(3)	17.3006(4)
b (Å)	18.3888(4)	19.3900(4)
c (Å)	24.5710(6)	20.7716(4)
α (deg)	94.772(2)	95.310(2)
β (deg)	109.004(2)	112.975(2)
γ (deg)	106.661(2)	99.330(2)
volume (Å^3)	6480.2(3)	6236.6(2)
Z	2	1
temp (K)	123(2)	123(2)
ρ_{calcd} (g cm^{-3})	1.724	1.721
GOF	1.068	1.033
R_1[a] ($I > 2\sigma(I)$)	0.0911	0.0655
wR_2[a]	0.2579	0.1870

[a] $R_1 = \Sigma||F_o|-|F_c||/\Sigma|F_o|$, $wR_2 = \{\Sigma[w(F_o^2 - F_c^2)]/\Sigma[w(F_o^2)^2]\}^{1/2}$.

Table S2. BVS values of metal and oxygen atoms of **I**.

P0	4.946	O16	2.018
Mn1	3.033	O17	1.832
Mn2	3.048	O18	1.960
Mn3	3.026	O19	1.862
Mn4	4.015	O20	2.019
Mo1	6.159	O21	1.822
Mo2	6.172	O22	2.021
Mo3	6.132	O23	1.812
Mo4	6.117	O24	1.997
Mo5	6.061	O25	1.731
Mo6	6.142	O26	1.918
Mo7	5.997	O27	1.862
Mo8	6.121	O28	2.003
Mo9	6.091	O29	1.937
O1	1.746	O30	1.943
O2	1.957	O31	1.983
O3	1.869	O32	1.928
O4	1.893	O33	1.877
O5	1.929	O34	1.954
O6	1.879	O35	1.965
O7	1.897	O36	1.940
O8	1.920	O37	1.911
O9	1.895	O38	1.788
O10	1.909	O39	1.484
O11	1.886	O40	1.788
O12	1.748	O41	1.944
O13	1.949	O42	1.822
O14	1.940	O43	2.023
O15	1.924		

Table S3. Selected bond lengths and angles in **I**.

	Bond lengths (Å)		
Mn1–O1	2.119	Mn3–O14	1.894
Mn1–O7	1.933	Mn3–O15	1.915
Mn1–O9	1.943	Mn3–O16	2.239
Mn1–O10	1.890	Mn4–O2	1.957
Mn1–O11	1.902	Mn4–O4	1.938
Mn1–O16	2.243	Mn4–O6	1.943
Mn2–O3	2.130	Mn4–O7	1.859
Mn2–O7	1.938	Mn4–O8	1.854
Mn2–O8	1.920	Mn4–O9	1.857
Mn2–O12	1.906	Mn1···Mn2	3.188
Mn2–O13	1.892	Mn1···Mn3	3.186
Mn2–O16	2.233	Mn1···Mn4	2.792
Mn3–O5	2.099	Mn2···Mn3	3.189
Mn3–O8	1.944	Mn2···Mn4	2.782
Mn3–O9	1.932	Mn3···Mn4	2.787
	Angles (°)		
Mn1–O7–Mn2	110.8	Mn4–O7–Mn1	94.8
Mn2–O16–Mn1	90.8	Mn4–O9–Mn1	94.5
Mn2–O8–Mn3	111.2	Mn4–O7–Mn2	94.2
Mn2–O16–Mn3	91.0	Mn4–O8–Mn2	95.0
Mn3–O9–Mn1	110.6	Mn4–O8–Mn3	94.4
Mn3–O16–Mn1	90.6	Mn4–O9–Mn3	94.7

Table S4. BVS values of metal and oxygen atoms of **II**.

P1	4.701	O13	1.774
Mn1	2.093	O14	1.788
Mn2	2.044	O15	1.759
Mn3	3.078	O16	1.769
Mo1	5.966	O17	1.788
Mo2	6.058	O18	2.031
Mo3	6.007	O19	1.950
Mo4	6.009	O20	2.017
Mo5	6.060	O21	1.884
Mo6	6.076	O22	2.097
Mo7	6.031	O23	1.948
Mo8	6.080	O24	1.940
Mo9	5.959	O25	1.917
O1	1.903	O26	1.970
O2	1.837	O27	1.927
O3	1.838	O28	1.981
O4	1.822	O29	1.959
O5	2.053	O30	1.793
O6	1.931	O31	1.745
O7	0.278	O32	1.793
O8	1.812	O33	1.919
O9	1.767	O34	2.042
O10	1.824	O35	1.960
O11	2.043	O36	1.948
O12	1.803	O37	1.668

Table S5. Selected bond lengths and angles in **II**.

Bond lengths (Å)	
Mn1–O1	2.273
Mn1*–O1	2.313
Mn1–O5	2.120
Mn1–O6	2.079
Mn1–O10	2.094
Mn1–O11	2.113
Mn2–O1	2.283
Mn2–O5	2.151
Mn2–O7	2.239
Mn2–O8	2.088
Mn2–O9	2.105
Mn2–O11	2.153
**Angles (°)**	
Mn1–O1–Mn1*	94.6
Mn1–O1–Mn2	92.6
Mn1*–O1–Mn2	93.3
Mn1–O5–Mn2	101.7
Mn1*–O11–Mn2	102.2

Table S6. Comparison of the $\{Mn^{III}_3Mn^{IV}O_3(OAc)_3\}$ core in **I** and other reported POM structures containing cubane cores.

Molecular Formula	Mn1···Mn4(Å)	Mn2···Mn4(Å)	Mn3···Mn4(Å)	Average $Mn^{III}\cdots Mn^{IV}$ (Å)	Ref.
$TPP_4[HMn^{III}_3Mn^{IV}(OAc)_3(B\text{-}\alpha\text{-}PMo_9O_{37})]$	2.792	2.782	2.787	2.787	This work
$[(CH_3)_2NH_2]_{5.33}H_2Mn^{II}_{0.33}[(\alpha\text{-}P_2W_{15}O_{56})Mn^{III}_3Mn^{IV}O_3(CH_3COO)_3]$	2.795	2.804	2.804	2.801	S8
$Na_{3.5}K_{2.5}[Mn^{III}_3Mn^{IV}O_3(CH_3COO)_3(A\text{-}\alpha\text{-}SiW_9O_{34})]$	2.749	2.937	2.945	2.877	S9
$Na_{3.5}K_{2.5}[Mn^{III}_3Mn^{IV}O_3(CH_3COO)_3(A\text{-}\beta\text{-}SiW_9O_{34})]$	2.810	2.817	2.881	2.836	S9

Table S7. Comparison of the $\{Mn_4O_{14}(H_2O)_2\}$ core in **II** and other reported representative manganese-containing Weakley-type structures.

Molecular Formula	$Mn1\cdots Mn1^*$(Å)	$Mn2\cdots Mn2^*$ (Å)	$(Mn2\cdots Mn2^*)/(Mn1\cdots Mn1^*)$	Mn1–O7(Å)	Ref.
$TPP_5H[\{Mn^{III}(acac)\}_2Mn^{II}_4(H_2O)_2(B\text{-}\alpha\text{-}PMo_9O_{34})_2]$	3.370	5.713	1.695	2.239	This work
$[Na_{11}(H_2O)_{25}]H[Mn_4(H_2O)_2(B\text{-}\alpha\text{-}GeMo_9O_{34})_2]$	3.297	5.600	1.699	2.173	S10
$K_{10}[Mn_4(H_2O)_2(PW_9O_{34})_2$	3.447	5.609	1.627	2.157	S11
$K_4Na_2[\{Ce(H_2O)_7\}_2Mn_4Si_2W_{18}O_{68}(H_2O)_2]$	3.253	5.598	1.721	2.248	S12
$[Ag(phen)_2]_6H_2[\{Mn(phen)\}_2Mn_4(H_2O)_2(\alpha\text{-}GeW_9O_{34})_2]$	3.415	5.593	1.638	2.216	S13

(a)

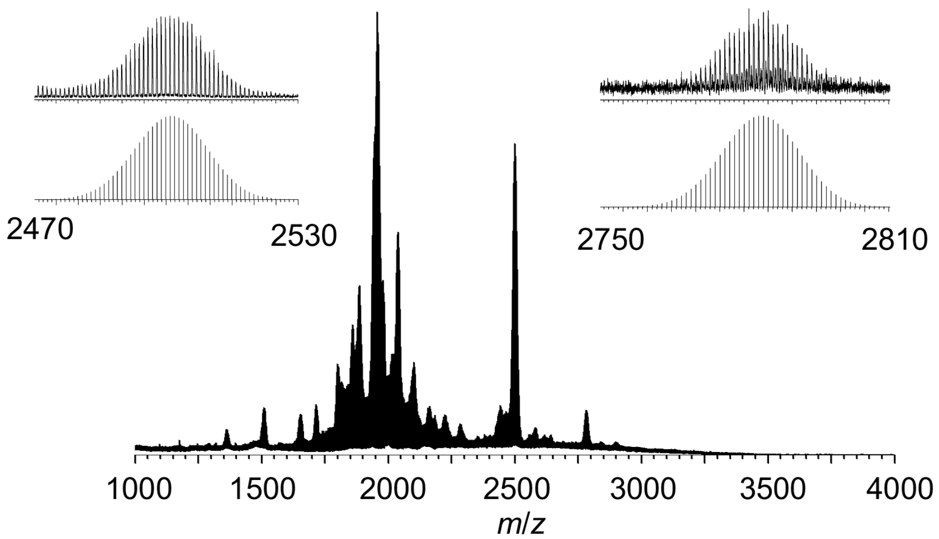

(b)

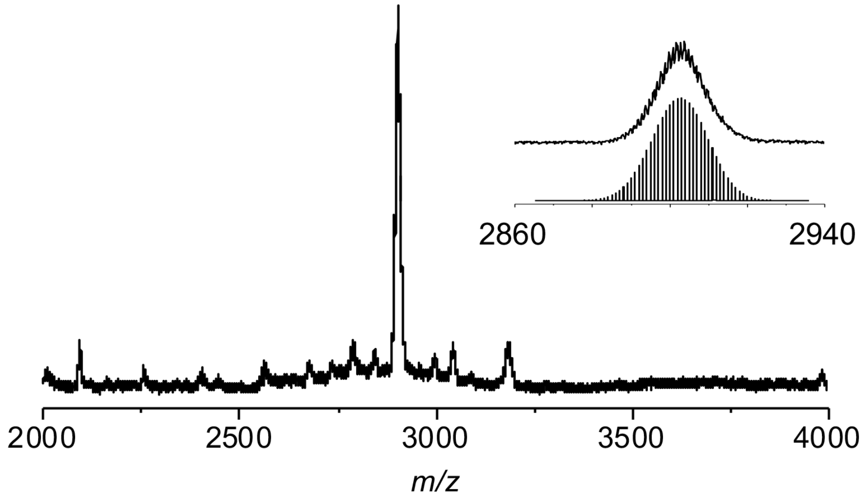

Fig. S1 (a) Negative-ion ESI-mass spectrum of the reaction solution of $TPP_3H_6[PMo_9O_{34}]$ and $Mn(OAc)_3$ in acetonitrile after stirring for 1 h. Insets: a spectrum in the *m/z* range 2470–2530 and a simulated pattern for $[TPP_2PMo_{12}O_{40}]^-$ and a spectrum in the *m/z* range 2750–2810, and a simulated pattern for $[TPP_3PMo_{11}MnO_{39}]^-$, respectively; (b) Negative-ion CSI-mass spectrum of the reaction solution of $TPP_3[PMo_9O_{31}(py)_3]$ (**PMo9-py**) and $Mn(OAc)_3$ in acetonitrile after stirring at 0°C for 2 h. Insets: a spectrum in the *m/z* range 2860–2940 and a simulated pattern for $[TPP_3H_1PMo_9O_{37}Mn_4(CH_3COO)_3]^-$.

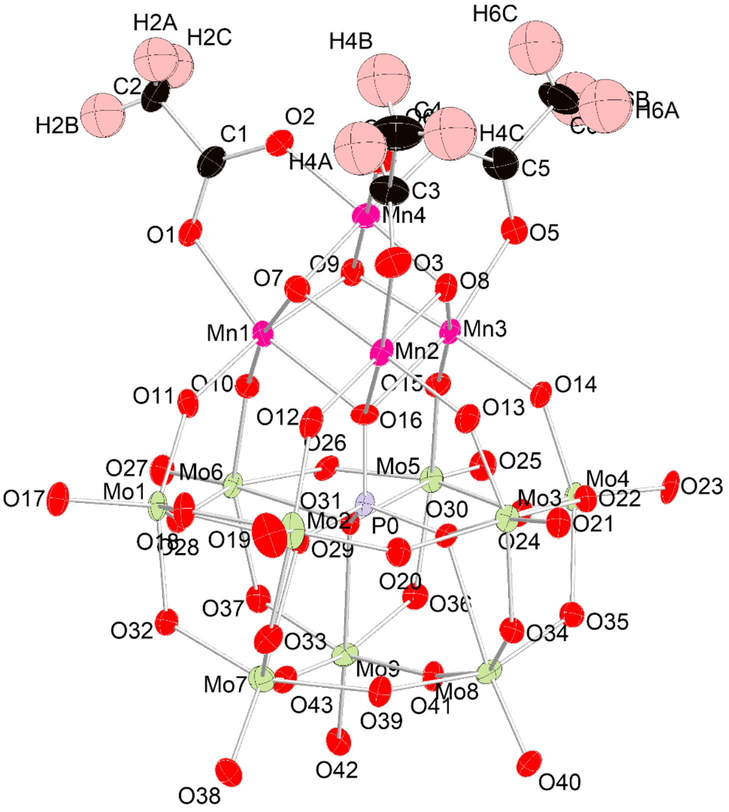

Fig. S2 ORTEP representation of the anion part of **I** with thermal ellipsoids drawn at the 50% probability level.

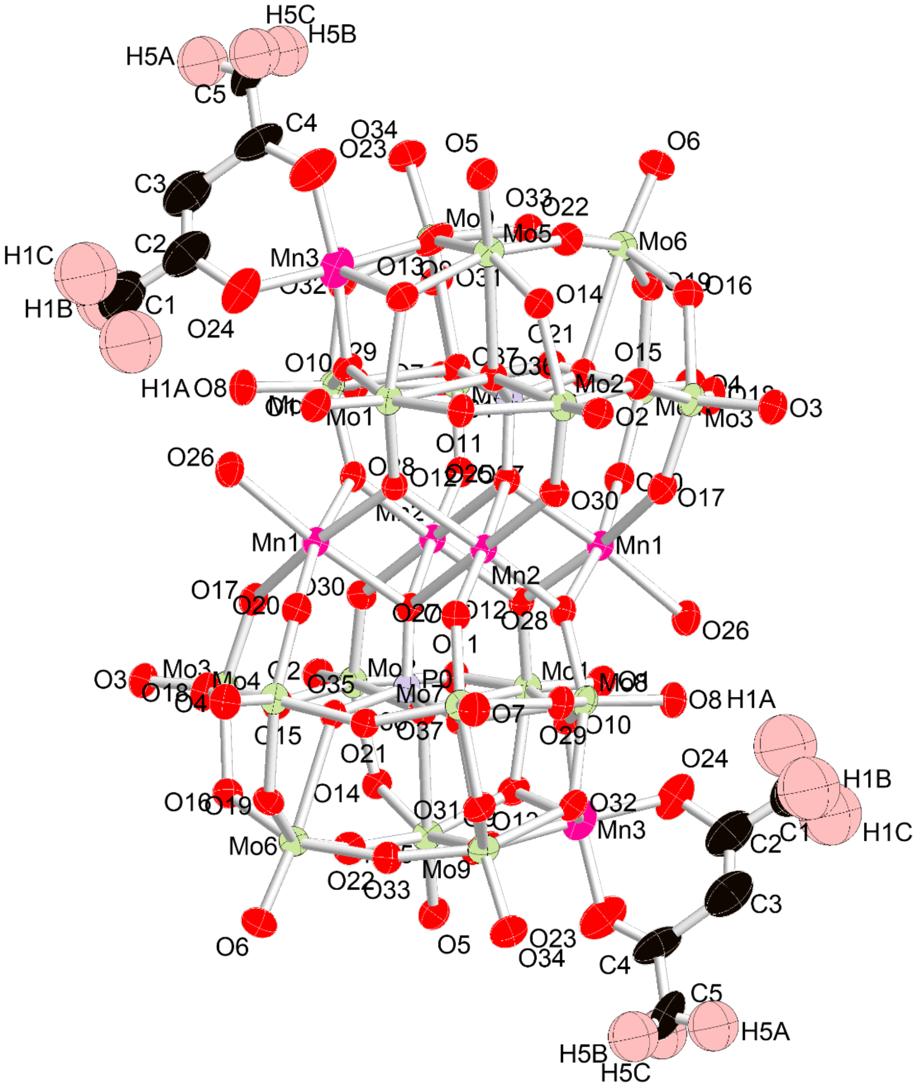

Fig. S3 ORTEP representation of the anion part of **II** with thermal ellipsoids drawn at the 50% probability level.

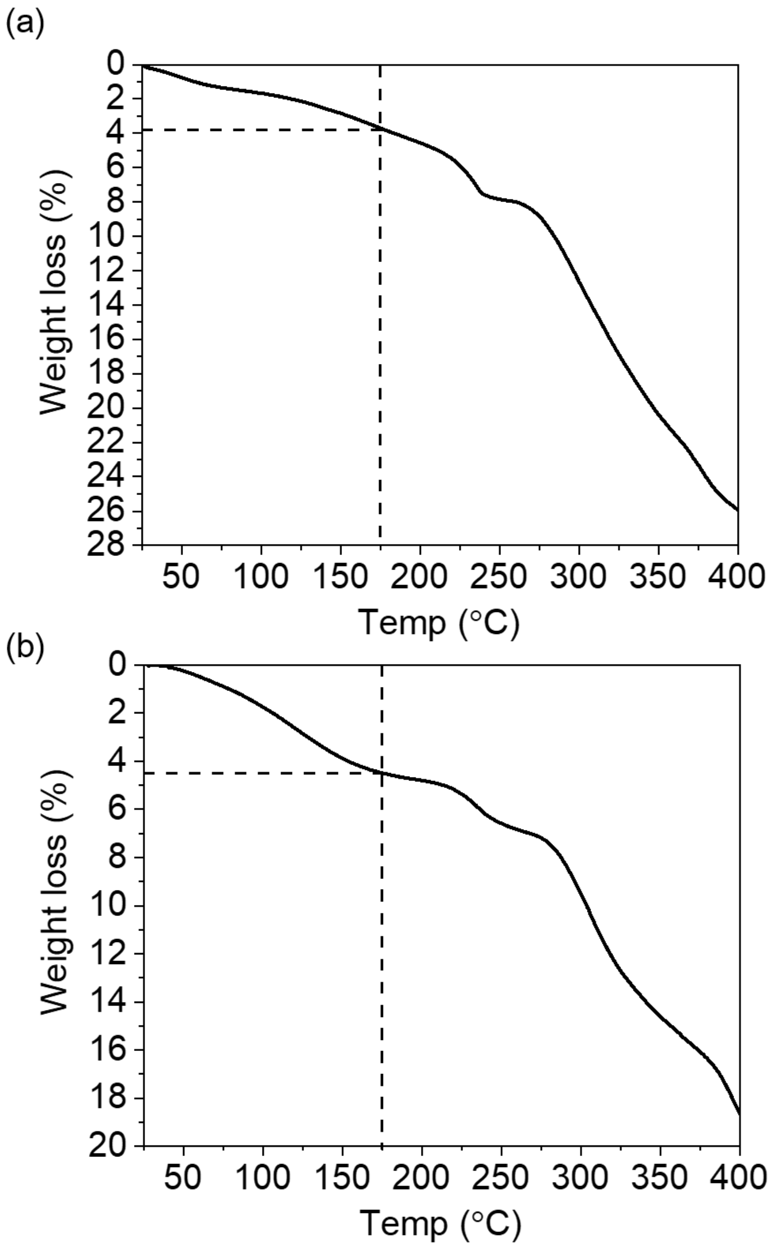

Fig. S4 TG curve of (a) **I** and (b) **II** (N_2 atmosphere).

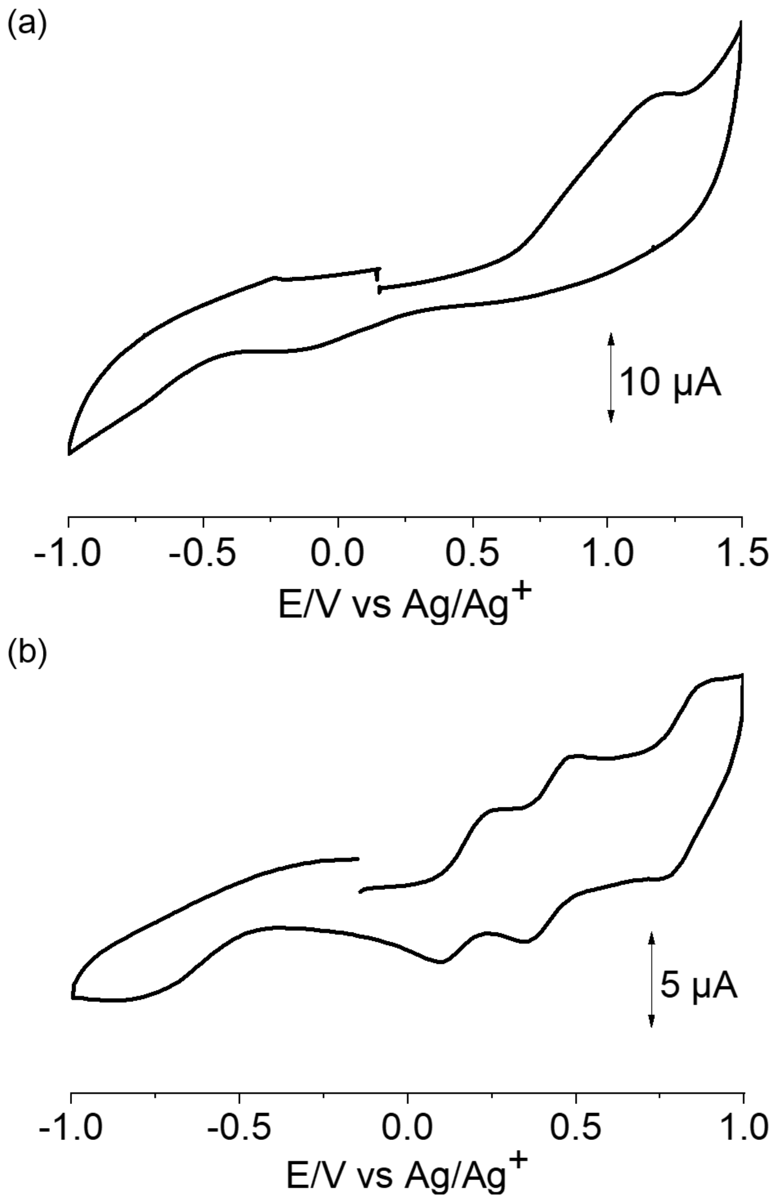

Fig. S5 Cyclic voltammogram of (a) **I** (1 mM), and (b) **II** (0.5 mM) in acetonitrile (100 mM $TBAClO_4$).

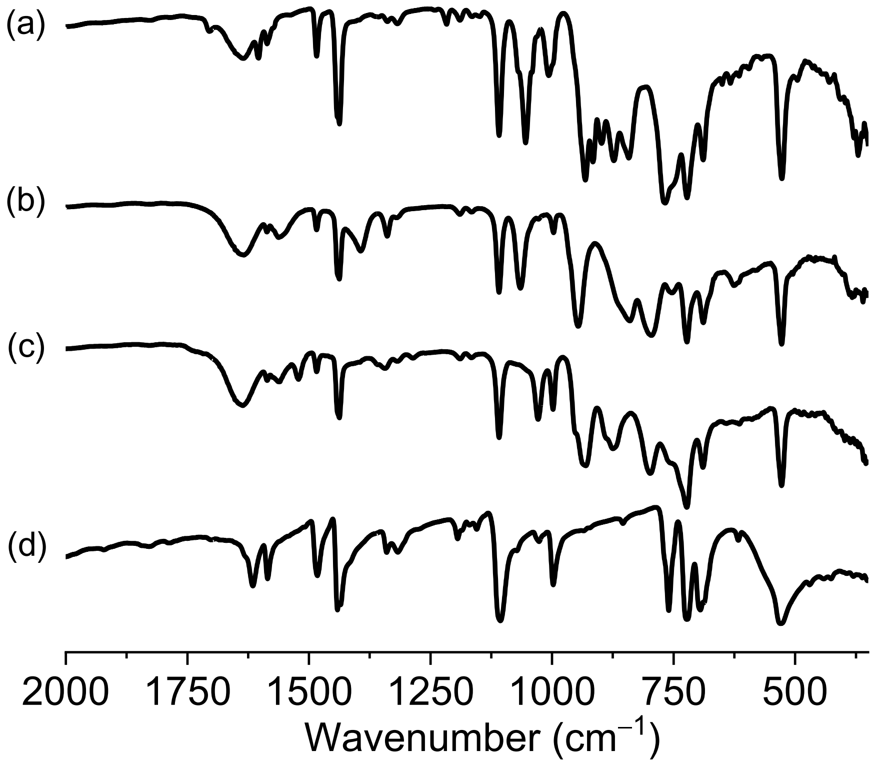

Fig. S6 IR spectrum of (a) **PMo9-py**, (b) **I**, (c) **II**, and (d) TPPBr.

Additional references

S1 Rigaku OD. *CrysAlis PRO*. Rigaku Oxford Diffraction Ltd, Yarnton, England (2018).

S2 L. J. Farrugia, *J. Appl. Crystallogr.*, 1999, **32**, 837.

S3 (a) G. M. Sheldrick, *Acta Cryst.,* 2008, **A64**, 112; (b) G. M. Sheldrick, *Acta Cryst*., 2015, **C71**, 3.

S4 P. van der Sluis and A. L. Spek, *Acta Crystallogr.*, 1990, **A46**, 194

S5 N. E. Brese and M. O'Keeffe, *Acta Crystallogr. Sect*., 1991, **B47**, 192.

S6 I. D. Brown and D. Altermatt, *Acta Crystallogr. Sect. B* 1985, **41**, 244.

S7 C. Li, N. Mizuno, K. Yamaguchi and K. Suzuki, *J. Am. Chem. Soc.*, 2019, **141**, 7687.

S8 X. Fang, M. Speldrich, H. Schilderb, R. Cao, K. P. O'Halloran, C. L. Hill and P. Kögerler, *Chem. Commun.*, 2010, **46**, 2760.

S9 R. Al-Oweini, B. S. Bassil, J. Friedl, V. Kottisch, M. Ibrahim, M. Asano, B. Keita, G. Novitchi, Y. Lan, A. Powell, U. Stimming and U. Kortz, *Inorg. Chem.,* 2014, **53**, 5663.

S10 S. Li, J. Zhao, P. Ma, J. Du, J. Niu and J. Wang, *Inorg. Chem.*, 2009, **48**, 9819.

S11 C. J. Gomez-Garcia, E. Coronado, P. Gomez-Romero and N. Casan-Pastor, *Inorg. Chem.,* 1993, **32**, 3378.

S12 W. Chen, Y. Li, Y. Wang and E. Wang, *Eur. J. Inorg. Chem.*, 2007, 2216.

S13 J. Wang, P. Ma, Y. Shen and J. Niu, *Cryst. Growth Des*., 2008, **8**, 3130.
